# Neurophysiology of the Developing Cerebral Cortex: What We Have Learned and What We Need to Know

**DOI:** 10.3389/fncel.2021.814012

**Published:** 2022-01-03

**Authors:** Heiko J. Luhmann

**Affiliations:** Institute of Physiology, University Medical Center Mainz, Mainz, Germany

**Keywords:** cerebral cortex, neurophysiology, development, rodent, methods

## Abstract

This review article aims to give a brief summary on the novel technologies, the challenges, our current understanding, and the open questions in the field of the neurophysiology of the developing cerebral cortex in rodents. In the past, *in vitro* electrophysiological and calcium imaging studies on single neurons provided important insights into the function of cellular and subcellular mechanism during early postnatal development. In the past decade, neuronal activity in large cortical networks was recorded in pre- and neonatal rodents *in vivo* by the use of novel high-density multi-electrode arrays and genetically encoded calcium indicators. These studies demonstrated a surprisingly rich repertoire of spontaneous cortical and subcortical activity patterns, which are currently not completely understood in their functional roles in early development and their impact on cortical maturation. Technological progress in targeted genetic manipulations, optogenetics, and chemogenetics now allow the experimental manipulation of specific neuronal cell types to elucidate the function of early (transient) cortical circuits and their role in the generation of spontaneous and sensory evoked cortical activity patterns. Large-scale interactions between different cortical areas and subcortical regions, characterization of developmental shifts from synchronized to desynchronized activity patterns, identification of transient circuits and hub neurons, role of electrical activity in the control of glial cell differentiation and function are future key tasks to gain further insights into the neurophysiology of the developing cerebral cortex.

## Introduction—What Are Our Tools?

In the last decade life sciences has shown a tremendous progress in developing and improving novel technologies. In cellular neurophysiology classical electrophysiological recording techniques and cellular imaging methods are very successfully combined with new tools in molecular biology and genetics to gain deeper insights into the function of single neurons and defined neuronal networks. Using the powerful Patch-seq technique, whole-cell patch-clamp recordings can be combined with morphological reconstructions, immunohistochemistry, and single-cell RNA-sequencing (scRNA-seq) to perform a multidimensional characterization of a single cell and to correlate the gene expression profile with its structural and functional properties (Cadwell et al., 2017; Scala et al., 2021). When combined with retrograde labeling, information on the projection patterns of single neurons can be added (Peng et al., 2021). Laser capture microdissection allows the transcriptomic characterization of subcellular compartments, such as soma vs. dendrite (Perez et al., 2021). Paired recordings from synaptically connected neurons combined with precise 3D reconstructions of the cells' morphology provided very detailed information on the function of local neuronal networks, e.g., a cortical column (Feldmeyer et al., 2018). With the vitro multi-patch approach 10–15 neurons can be simultaneously patch-clamp recorded, intracellularly labeled and subsequently analyzed in their synaptic connectivity (Peng et al., 2019). Functional connectivity maps at a larger scale, but with less temporal and spatial precision, can be obtained in brain slices with caged glutamate laser-scanning photostimulation (Meng et al., 2020).

Beside these advances in *in vitro* neurophysiology, *in vivo* recording, and stimulation techniques became also very powerful. Two-photon microscopy can be used to accomplish whole-cell membrane potential recordings of fluorescently labeled and genetically defined neurons in awake head-fixed mice while performing specific behavioral tasks (Petersen, 2017). Juxtacellular stimulation and recording of single cells in head-fixed or freely moving rats allows to study the role of single neurons in cognition and behavior (Houweling and Brecht, 2008; Tang et al., 2014). The number of simultaneously recorded neurons increased over the last decade from a few hundreds to a few thousands (Steinmetz et al., 2019; Perez-Prieto and Delgado-Restituto, 2021) and we may soon approach the neurophysiologist's dream (or nightmare?) to “concurrently record electrical spikes from every neuron in a mammalian brain” (Kleinfeld et al., 2019). To reach this aim, close cooperations with other scientific fields are already on-going (Jun et al., 2017; Garcia-Etxarri and Yuste, 2021). Material sciences and electronic engineering will foster the development of biocompatible multi-electrode arrays with very high number of electrodes, which can be used for recordings of single units and focal electrical stimulation (Jun et al., 2017; Kleinfeld et al., 2019; Perez-Prieto and Delgado-Restituto, 2021). Recently, chronic single unit recordings could be obtained from freely moving rats over up to 4 months with Neuropixel electrodes (Luo et al., 2020). These technological developments will also have an impact on experimental and clinical approaches of deep brain stimulation and brain-machine interfaces.

In the last decade subcellular, cellular, meso-scale, and large-scale imaging methods also showed tremendous technological progress. Genetically encoded calcium indicators, mostly from the GCaMP family, are powerful tools to monitor neuronal activity in small networks up to large-scale dynamics in behaving animals (Cardin et al., 2020; Linden et al., 2021; Ren and Komiyama, 2021). The large majority of these *in vivo* studies are restricted to upper neocortical regions because of technical limitations (Yang and Yuste, 2017). However, red-shifted fluorescent calcium indicators (Tischbirek et al., 2017) and three-photon microscopy (Takasaki et al., 2019) now enables imaging of deep cortical layers with single-cell resolution. Longitudinal calcium imaging from cortical and also deep brain structures can be obtained in freely behaving mice with the (wireless) miniature fluorescence microscope (miniScope) technology (Barbera et al., 2019; Zhang et al., 2019), which also allows recording of neuronal activity at single-cell resolution. Recently high-resolution two-photon calcium imaging of both cortical hemispheres have been performed in awake head-fixed mice (Barson et al., 2020; Cardin et al., 2020). This powerful method allows for the first time the analysis of the entire cortical mantle with cellular resolution. However, calcium imaging still has a number of disadvantages when compared to cellular neurophysiology. Electrophysiological recordings with sub-millisecond resolution provides high temporal information on the spiking output of the neuron, whereas calcium imaging cannot directly monitor the output, but rather monitors the increase in [Ca^2+^]_i_ following (subthreshold) synaptic activation and internal state changes (West et al., 2001). A recent study in the visual cortex of awake mice using the genetically encoded calcium indicator GCaMP6f and extracellular recordings with silicon probes demonstrated the advantages and disadvantages of both methods (Siegle et al., 2021). While electrophysiological recordings showed a higher fraction of units with stimulus-driven activity, GCaMP6f calcium imaging revealed a higher selectivity (sharper tuning) among responsive neurons. Another *in vivo* study in the visual cortex of awake transgenic mice expressing GCaMP6s or GCaMP6f demonstrated that <10% and 20–30%, respectively, of isolated single spikes were detectable with calcium imaging (Huang et al., 2021). Critical assessment of calcium imaging data (Rupprecht et al., 2021) and the development of new calcium indicators with fast kinetics (such as jGCaMP8) may solve some of these problems (Zhang et al., 2021).

Subthreshold synaptic inputs are very difficult to detect with somatic calcium signals. Genetically encoded voltage indicators (GEVIs) can monitor subthreshold transmembrane potential dynamics *in vitro* and *in vivo* (Bando et al., 2019). The classical voltage-sensitive dyes developed over the last 30 years were most valuable in elucidating cortical function (Grinvald et al., 2016). The GEVIs have the advantage that they can be targeted to specific cell types and when combined with 2-photon excitation allows imaging of membrane potential dynamics with single-cell resolution in the cerebral cortex *in vivo* (Bando et al., 2019). GEVIs can be also used at the subcellular level, e.g., to study action potential generation at the axon initial segment or transmitter release at the presynaptic terminal (Panzera and Hoppa, 2019).

Beside this progress in cellular electrophysiological and imaging techniques a number of novel genetic and molecular biological methods became most valuable tools to manipulate gene expression and activity of defined single cells during distinct time points of development. Targeted gene delivery via *in vivo* (e.g., *in utero*) electroporation (De Vry et al., 2010), directed genetic manipulations by CRISPR-based technology (Kampmann, 2020), viral tools for specific cell and circuit manipulation (Vormstein-Schneider et al., 2020), Designer Receptors Exclusively Activated by Designer Drugs (DREADD)-based chemogenetic tools (Roth, 2016), and optogenetics (Deisseroth, 2015) are used to activate or silence specific neurons. Some of these methods, especially optogenetics, become even more powerful when combined with *in vivo* targeted whole-cell recordings in behaving animals (Gasselin et al., 2021) or with miniScope technology in freely moving mice (de Groot et al., 2020).

These impressive methodological developments offer the chance to booster interdisciplinary cooperations between experts in cellular electrophysiology, molecular biology, and biostatistics or biomathematicians, and as in the past, physicists are always welcome. Does that mean that the times, that a cellular neurophysiologist is sitting for 6–12 h alone at his/her *in vitro* or *in vivo* set-up, obtaining all relevant data on-line and analyzing the data with rather simple statistics and mathematics programs is over?

The next three sections will focus on the cellular neurophysiology of the developing cerebral cortex in rodents. Since the function of individual neurons is not only determined by its intrinsic properties, but also by its synaptic inputs, early cortical network activity will be also briefly discussed.

## The Challenges of Studying the Neurophysiology of the Developing Cerebral Cortex in Rodents

Everybody who studies the cellular and network properties of the cerebral cortex in newborn rodents faces a number of challenges. In mice and rats (and in many other mammals such as ferrets), the cerebral cortex at birth has not yet reached its mature six-layered structure. Neurons are still migrating to their final location in layer (L) 2 and L3, perhaps even L4, and these layers are formed until postnatal day (P) 2–3. This migration process, as neuronal proliferation and differentiation (for review Uhlen et al., 2015), is controlled by spontaneous intracellular calcium transients (Bando et al., 2016). Thus, the neocortical network of a newborn rodent changes its layered architecture on a daily basis!

This add-on process in neocortical layering is accompanied by the reorganization of an early transient cortical network, which at birth consists of the marginal zone (later L1), the cortical plate (later L2–L6) and the subplate (for review Kanold and Luhmann, 2010; Hoerder-Suabedissen and Molnár, 2015). At late embryonic and early postnatal stage thalamocortical afferents form functional glutamatergic synapses with subplate neurons, which play important roles in synaptic plasticity and synchronized network activity of the developing cortical network (for review Colonnese and Phillips, 2018; López-Bendito, 2018; Luhmann et al., 2018; Molnár et al., 2020). Between P1 and P2, these thalamocortical fibers disconnect from the subplate and innervate their final targets in L4 and L5/6. Another transient thalamocortical circuit is assembled by infragranular somatostatin (SST) expressing interneurons which innervate parvalbumin (PV) and pyramidal neurons. This early thalamocortical-intracortical circuit disappears by the end of the first postnatal week and is important for the development of thalamic feedforward inhibition via PV interneurons (Tuncdemir et al., 2016). Using transgenic mouse lines Marques-Smith et al. identified in the neonatal mouse somatosensory a transient and activity-dependent early reciprocal circuit cortex between L5b SST-positive interneurons and L4 spiny stellate cells (Marques-Smith et al., 2016). Thus, the newborn rodent cortex exhibits a number of transient circuits and changes its thalamic activation pattern within 3–4 days.

Beside subplate cells, other neocortical neurons also fulfill important roles in early cortical development and are only transiently present during peri-/neonatal stages. Cajal-Retzius neurons in the marginal zone/L1 control a number of important developmental processes and mostly disappear by programmed cell death (apoptosis) during the first postnatal days after termination of neuronal migration (Causeret et al., 2021). Beside subplate and Cajal-Retzius neurons, glutamatergic and GABAergic neurons also die to a large extent by apoptosis during the first postnatal week (with a peak at ~P7) (Causeret et al., 2018). Notably, this apoptosis process is strongly regulated by electrical activity (Blanquie et al., 2017; Riva et al., 2019). Beside the activity-dependent control in overall cell number, the axonal and dendritic morphology and synaptic connectivity of single cortical neurons is also regulated by early activity (Callaway and Borrell, 2011; Malyshevskaya et al., 2013; Grant et al., 2016) (for review Leighton and Lohmann, 2016). Defined subpopulations of GABAergic interneurons control spontaneous cortical activity patterns and play central roles in the early postnatal development of the cerebral cortex (Leighton et al., 2021). During the first two postnatal week, vasocative-intestinal-peptide and somatostatin interneurons undergo developmental transitions in their cortical function and their processing of sensory information (Kastli et al., 2020). Well-connected early generated interneurons with relatively mature electrophysiological and morphological properties exert a strong influence on cortical activity and ablation of these neurons disturbs spontaneous activity and inhibitory synapse formation (Wang et al., 2019). Using longitudinal *in vivo* calcium imaging of somatosensory cortical activity in non-anesthetized mouse pups, Duan et al. convincely demonstrated that developmental network patterns in interneurons and pyramidal cells are essential for the assembly of neocortical circuits and for the control of interneurons' cell death (Duan et al., 2020). Using *in vivo* imaging in the developing mouse barrel cortex, Modol et al. show that PV interneurons form transient periphery-driven and experience-dependent patches of correlated activity (Modol et al., 2020). Embryonic disturbances in interneuron generation and cortical network integration cause a transient dysfunction with long-term behavioral consequences (Magno et al., 2021). Thus, during the first two postnatal weeks the rodent cerebral cortex is characterized by the deconstruction of transient neuronal networks and simultaneously by the construction of its characteristic six-layered, columnar architecture, intracortical microcircuits, corticothalamic connections, and corticocortical connections.

Spontaneous and sensory evoked activity, monitored by calcium imaging *in vivo*, has been not only observed in neurons of the developing cerebral cortex (Rochefort et al., 2009; Yuryev et al., 2018), but also in astrocytes (Wang et al., 2006). In the embryonic ventricular zone spontaneous calcium waves propagate through radial glia cells and modulate proliferation (Weissman et al., 2004). Astrocytes in newborn mouse neocortical slices reveal spontaneous ultraslow (~2 per hour) and very long (~8 min) sodium fluctuations that are largely restricted to the first postnatal week (Felix et al., 2019). The mechanisms underlying this slow activity and its functional role are currently unknown. Spontaneous activity controls myelination since oligodendrocytes preferentially myelinate electrically active axons via axo-glial interactions leading to local calcium rises in glial cell processes (Wake et al., 2015). *In vitro* data indicate that patterned neuronal activity promotes the survival of oligodendrocytes (Gary et al., 2012) and that GABA released from inhibitory neurons controls myelination and internode length thereby tuning the conduction velocity (Hamilton et al., 2017). Vice versa, oligodendrocyte precursor cells not only respond to, but also modulate neuronal network function, demonstrating a bidirectional glia-neuron cross-talk (Sakry et al., 2014).

This developmental reorganization in cortical structure is accompanied by four neurophysiological processes, which have a strong impact on early cortical function and development. As in the hippocampus (Ben-Ari, 2014), the cerebral cortex also shows a developmental shift in GABA action from depolarizing/excitatory to hyperpolarizing/inhibitory (for review Kirmse et al., 2018; Kilb, 2020). This shift in GABA action differs between *in vitro* and *in vivo* preparations as shown by optogenetic studies (Valeeva et al., 2016). Electrophysiological differences between *in vitro* and *in vivo* preparations have been further reported following the deletion of NKCC1, the primary chloride inward transporter (Graf et al., 2021). *In vivo* reports (Kirmse et al., 2015; Murata and Colonnese, 2020) and modeling studies (Lombardi et al., 2021) provide a more complex picture of the developmental shift in GABA action, especially when on-going spatiotemporal interactions between GABAergic and glutamatergic inputs are taken into account (for review Kilb, 2021). It is not the aim of this short review to summarize the current data on this topic, but it is well-accepted that the intracellular chloride concentration is regulated by development, various molecular factors, and neuronal activity (Kaila et al., 2014; Watanabe and Fukuda, 2015; Virtanen et al., 2020, 2021). Thus, the action and function of the important neurotransmitter GABA, and also glycine and taurine (for review Kilb and Fukuda, 2017), changes dramatically during the first postnatal week in rodent cortex!

The second developmental change in cortical function is the transition from electrical coupling via gap junctions to mostly chemical synaptic transmission (Valiullina et al., 2016; Yao et al., 2016). During embryonic and early postnatal development, gap junctions play important roles in various developmental processes (Allene and Cossart, 2010; Niculescu and Lohmann, 2014). Yu et al. found strong electrical coupling between clonally related excitatory neurons in columnar manner forming the functional template of ontogenetic columns (Yu et al., 2012). Lineage-related electrical coupling has been also demonstrated between interneurons of the same subtype over an extended period of time and across a range of distances (Zhang et al., 2017). At later stages electrical synapses may interact with chemical synapses (Pereda, 2014). Neonatal connexin 26 removal impairs neocortical development and leads to elevated anxiety (Su et al., 2017). With maturation intercellular communication via gap junctions largely disappears during the second postnatal week and remains restricted to specific cell types, mostly interneurons (Connors, 2017), or cell compartments as axo-axonal coupling (Traub et al., 2004). Thus, intercellular functional interactions shift from electrical coupling to chemical synaptic transmission!

The third developmental change in cortical function is the transition from highly synchronized network activity during the late prenatal/early postnatal period to a more desynchronized state at later stages. Using two-photon calcium imaging of the barrel cortex *in vivo*, Mizuno et al. demonstrated that during the first postnatal week L4 neurons within one barrel show synchronized spontaneous activity (Mizuno et al., 2018). This patchwork activity pattern disappeared during the second postnatal week when L4 neurons fired asynchronously within one barrel. Several spontaneous network activity patterns (cortical early network oscillations, spindle bursts/delta brushes, early gamma oscillations, cortical giant depolarizing potentials, spontaneous activity transients, and others) have been characterized in the neonatal cerebral cortex of various mammalian species, from mouse to human (Colonnese and Khazipov, 2012; Luhmann et al., 2016; Luhmann and Khazipov, 2018; Molnár et al., 2020). For some of these patterns, the subplate and other transient cortical circuits play a central role (Kanold and Luhmann, 2010; Colonnese and Phillips, 2018; Luhmann et al., 2018). Beside these developmental changes in the pattern of spontaneous activity, cortical processing of sensory evoked activity undergoes a similar transition from bursting in pre- and neonatal stages to continuous “adult-like” activity during further development (Colonnese et al., 2010). Thus, during early development the neocortex shifts from a highly synchronized to a more desynchronized state!

The fourth developmental change in cortical function is the gradual innervation of the cortical layers by ascending neuromodulatory systems, such as the cholinergic, serotonergic, dopaminergic, and the noradrenergic system. Although the subplate receives functional neuromodulatory inputs already at early stages (Hanganu and Luhmann, 2004; Dupont et al., 2006; Hanganu et al., 2009), cortical layers 2 to 6 are only gradually innervated in an inside first—out side last sequence (Calarco and Robertson, 1995; Mechawar and Descarries, 2001). The emergence of the neuromodulatory inputs accompanies the developmental switch from bursting to continuous desynchronized activity (Colonnese et al., 2010) and the developmental changes in vigilance states and active movements (Mukherjee et al., 2017; Dooley et al., 2020; Glanz et al., 2021). Thus, these neuromodulatory systems have a progressively stronger influence on spontaneous and sensory evoked cortical function during the early postnatal period.

These dramatic structural and functional changes in the rodent developing cortex represent a major problem for everybody studying early neocortical development in mice and rats, because one should not (or better cannot) merge data from different postnatal days. The neocortex of a P0 mouse is different from that of a P1 mouse, and that differs from a P2 mouse! In a careful and detailed study, one should either focus on one single postnatal day (Mojtahedi et al., 2021) or form groups that differ in age not more than 1–2 days (Yang et al., 2009; Shen and Colonnese, 2016).

## Neurophysiology of the Developing Cerebral Cortex: What We Have Learned

Although recording techniques from freely moving rodents are important and become increasingly more powerful, such recordings from newborn rodents are only of limited value, because newborn mice and rats do not actively move so much during the first 10 postnatal days (van der Bourg et al., 2017). However, newborn rodents are exposed to a variety of sensory stimuli (e.g., from the proprioceptive and probably vestibular system) and from passive interactions with the mother and littermates. Sensory feedback from passive stimulation by littermates trigger cortical activity (Akhmetshina et al., 2016). Furthermore, spontaneous self-generated movements (myoclonic twitches) can be already observed very early and play an important role in the development of the sensorimotor system (Inacio et al., 2016; Dooley et al., 2020; Gomez et al., 2021). Head-fixation of newborn rodents allows simultaneous recordings of large-scale neuronal activity and spontaneous movements of the animal's snout including the whiskers, the forelimbs, hindlimbs, and the tail using electromyography (EMG) or multiple video camera monitoring (Dooley and Blumberg, 2018; Glanz et al., 2021; Gomez et al., 2021). Neuronal structures involved in the generation or modulation of spontaneous movements in newborns can be studied with imaging or multi-electrodes recording techniques. Central pattern generator (CPG) networks in spinal cord, brainstem, thalamus, motor cortex, and also cerebellum are of pivotal interest (Colonnese and Khazipov, 2012; Blumberg et al., 2013; Luhmann et al., 2016) and as many brain regions as possible should be recorded simultaneously in awake animals. CPGs and motor-related networks may trigger spontaneous movements, which subsequently activate the sensory system, thereby promoting the spatio-temporal binding of developing neuronal networks in the sensory-motor system. In the somatosensory and motor cortex of newborn rodents spindle burst and gamma oscillations mediate this binding process (Khazipov et al., 2004, 2013; Yang et al., 2009, 2013; Minlebaev et al., 2011; An et al., 2014; Luhmann and Khazipov, 2018; Dooley et al., 2020; Glanz et al., 2021).

Spindle burst and gamma oscillatory activity has been also demonstrated in the visual cortex of rodents before eye opening (Ackman and Crair, 2014; Colonnese and Phillips, 2018). Spindle bursts in the visual cortex are triggered by retinal bursts (Hanganu et al., 2006; Colonnese and Khazipov, 2010) and are modulated by the cholinergic system (Hanganu et al., 2006; Ackman et al., 2012). Spontaneous activity generated in the cochlea before hearing onset controls the development of central auditory pathways (Tritsch et al., 2007; Wang et al., 2015), but the function of spindle bursts and gamma oscillations in the auditory cortex of newborn rodents is less clear as compared to those in the visual and somatosensory system.

Nested gamma spindle bursts have been demonstrated in the prefrontal–hippocampal network of the neonatal rat (Brockmann et al., 2011; Bitzenhofer et al., 2015) and cholinergic projections facilitate this oscillatory coupling by activation of muscarinic acetylcholine receptors (Janiesch et al., 2011). In neonatal mice optogenetic activation of channelrhodopsin-expressing pyramidal neurons in L2/3, but not L5/6, of the prefrontal cortex boost oscillatory activity in beta–gamma frequency bands (Bitzenhofer et al., 2017).

In summary, spontaneous and sensory evoked activity pattern recorded in sensory, motor and prefrontal cortex of newborn rodents share many similarities and most likely fulfill similar functions. It is not surprising that these activity patterns also occur in the cerebral cortex of preterm human babies, when the cortex resembles in its structure and function the cortex of a newborn rodent (Colonnese et al., 2010; Luhmann and Fukuda, 2020). The activity patterns recorded in different cortical areas of preterm human babies are termed spindle bursts, delta brushes, *tracé discontinue, tracé alternant*, and spontaneous activity transients (SAT) (Vanhatalo and Kaila, 2006; Milh et al., 2007; Colonnese et al., 2010; Chipaux et al., 2013; Koolen et al., 2016) and serve as biomarkers for the cognitive and motor development of the child (Iyer et al., 2015; Tokariev et al., 2019; Moghadam et al., 2021).

Over the last decade we got deeper insights into the functional roles of these early cortical activity patterns. During embryonic stages spontaneous activity arising from the thalamus regulates coordinate cortical sensory area patterning and drives the emergence of functional cortical maps (Moreno-Juan et al., 2017; Anton-Bolanos et al., 2018, 2019; Martini et al., 2021). In newborn rodent cortex spontaneous and sensory evoked cortical activity often locally synchronizes column-like networks resembling early topographic cortical maps (Yang et al., 2013; Kummer et al., 2016). At perinatal stage this columnar cortical activity (“columnar burst,” Colonnese et al., 2010) is mediated by the activation of a local network in the subplate (Dupont et al., 2006; Hanganu et al., 2009). Removal of the subplate in the neonatal rat barrel cortex reduces spindle burst activity and prevents the development of the thalamocortical barrel field patterning (Tolner et al., 2012). Li et al. have demonstrated that not only the development of barrel-related columns, but also the emergence of L4 depends on early activity, specifically on glutamate released from thalamocortical afferents (Li et al., 2013).

In a number of elegant studies Hanganu-Opatz and coworkers demonstrated how genetic or experimentally induced manipulations in the oscillatory network activity in neonatal prefrontal cortex results in long-term functional and behavioral deficits (Chini and Hanganu-Opatz, 2021). In a mouse model of mental disorders, Chini et al. demonstrated an early-emerging prefrontal network dysfunction that subsequently gives rise to cognitive deficits. They show that this deficiency can be rescued by minocycline administration, thus identifying a potential biomarker (Chini et al., 2020). Bitzenhofer et al. manipulated early activity in the prefrontal cortex of neonatal mice, resulting in disruption of coordinated patterns of electrical activity, excitation-inhibition imbalance, and impaired cognitive abilities at adult age. Thus, prefrontal activity during development is critical for adult network function and behavioral performance (Bitzenhofer et al., 2021).

## The Open Questions and Future Tasks

This last section provides a personal viewpoint on the open questions and tasks that should be addressed in the third decade of the 21st century to gain further insights into the neurophysiology of the developing cerebral cortex. If possible, these studies should be performed *in vivo* in non-anesthetized animals.

1. Study with cell resolution recording and imaging techniques the large-scale interactions between different cortical areas (sensory, association, prefrontal, motor) and subcortical regions in pre- and early postnatal development. What are the underlying mechanisms of local vs. global interactions and what are their functions? Define the relationship and the role of local vs. propagating activity.
2. Identify at different developmental stages the interactions between spontaneous, movement related and sensory evoked activity. Characterize the developmental shift from synchronized to desynchronized network activity in sensory, prefrontal, and motor system including subcortical regions.
3. What is the functional role of early cortical activity patterns in shaping the pre- and early postnatal cortex at micro- (e.g., spines), meso- (e.g., microcircuitry), and macroscale (e.g., cortico-cortical connections)?
4. Determine the dynamic spatio-temporal action of GABA and glycine during earliest (prenatal) stages of development under non-anesthetized *in vivo* conditions and by the use of non-invasive techniques?
5. Characterize the role of neuromodulators (e.g., acetylcholine, noradrenaline, serotonin) on cortical neurophysiology at different developmental stages.
6. Define and elucidate the emergence of cognitive functions, e.g., learning and memory, spatial navigation, social interactions.
7. Characterize the role of distinct populations of early generated GABAergic interneurons (Wang et al., 2019) and identify and manipulate hub neurons in developing neocortical networks (cf. hub neurons in immature hippocampal networks Bonifazi et al., 2009; Picardo et al., 2011; Bocchio et al., 2020).
8. In their seminal review article Connors and Gutnick described in the rodent cerebral cortex three distinct functional cell types based on their intrinsic firing pattern (Connors and Gutnick, 1990): regular spiking, fast spiking and bursting neurons. Over the last 30 years we learned that cortical neurons, especially interneurons, can be subdivided into many more cell type classes (DeFelipe et al., 2013). A recent single-cell transcriptomic analysis of the mouse primary motor cortex identified over 56 neuronal cell types (Yao et al., 2021). Kriegstein and coworkers recently characterized 138 neocortical cell type clusters throughout the second trimester of human development and demonstrated that “gene-expression patterns are highly dynamic across cortical regions” and “borders between clusters are fluid” (Bhaduri et al., 2021). Since it is well-established that the firing pattern of neocortical neurons *in vivo* is strongly regulated by the action of modulatory systems (Steriade, 2001a), cell classifications based on firing patterns may be also more “fluid” as estimated by experimental protocols used in mostly *in vitro* slice preparations. Thus, cell type classifications in (developing) cerebral cortex should take technical limitations (c.f. Steriade, 2001b) and dynamic changes into account.
9. Identify the mechanisms and the functional role of activity-dependent interactions between neurons and astrocytes, oligodendrocytes, radial glia, and microglia in perinatal cortex. How do certain spontaneous activity patterns promote myelination (for review Zuchero and Barres, 2013)? How does neuronal activity influence the development of the tri-partite synapse (for review Dallerac et al., 2018) and “quad-partite” synapse including microglia (for review Schafer et al., 2013)?
10. Characterize the neuro-glia-vessel communication in perinatal cortex (Segarra et al., 2018) and identify a potential role of early spontaneous and sensory evoked activity in cortical vascularization and development of the blood-brain barrier (Whiteus et al., 2013).
11. Critically evaluate the use of rodent models in understanding early cortical development in humans (Luhmann and Fukuda, 2020) and whether rodents fulfill criteria to study preclinical manifestations of human diseases (Al Dahhan et al., 2019).
12. Organoids may become most valuable models for early cortical development in humans (Trujillo et al., 2019; Chan et al., 2021). However, it needs to be studied whether important cortical circuits present in pre- and neonatal human cortex are also present in organoids (e.g., the fetal subplate circuits Kostovic, 2020; Kostovic et al., 2021). Further, it remains to be studied in more detail whether the spontaneous activity patterns present in pre- and neonatal human cortex (Stjerna et al., 2012; Omidvarnia et al., 2014; Koolen et al., 2016) can be also demonstrated in human organoids.
13. Complement experimental studies with computational modeling of neocortical circuit development (Richter and Gjorgjieva, 2017; Shenoy and Kao, 2021).
14. The Introduction gave a brief overview on the recent technological developments. However, as Frégnac stated in his review we may face “dangers of letting technology-driven—rather than concept-driven—strategies shape the future industrialization of neuroscience through the rapid emergence of very-large-scale data-mining initiatives” (Frégnac, 2017).

## Author Contributions

The author confirms being the sole contributor of this work and has approved it for publication.

## Funding

HL was supported by the Deutsche Forschungsgemeinschaft.

## Conflict of Interest

The author declares that the research was conducted in the absence of any commercial or financial relationships that could be construed as a potential conflict of interest.

## Publisher's Note

All claims expressed in this article are solely those of the authors and do not necessarily represent those of their affiliated organizations, or those of the publisher, the editors and the reviewers. Any product that may be evaluated in this article, or claim that may be made by its manufacturer, is not guaranteed or endorsed by the publisher.
